# Synthesis of zinc oxide nanostructures on graphene/glass substrate by electrochemical deposition: effects of current density and temperature

**DOI:** 10.1186/1556-276X-9-609

**Published:** 2014-11-11

**Authors:** Nur Ashikyn Hambali, Hafizal Yahaya, Mohamad Rusop Mahmood, Tomoaki Terasako, Abdul Manaf Hashim

**Affiliations:** 1 Malaysia-Japan International Institute of Technology, Universiti Teknologi Malaysia, Jalan Semarak, Kuala Lumpur 54100, Malaysia; 2 Faculty of Electrical Engineering, Universiti Teknologi MARA, Shah Alam, 40450 Selangor, Malaysia; 3 Graduate School of Science and Engineering, Ehime University, 790-8577 Ehime, Japan

**Keywords:** Electrochemical deposition, Graphene, Nanorod, Zinc oxide, Solar cell, Hybrid structure

## Abstract

The electrochemical growth of zinc oxide (ZnO) nanostructures on graphene on glass using zinc nitrate hexahydrate was studied. The effects of current densities and temperatures on the morphological, structural, and optical properties of the ZnO structures were studied. Vertically aligned nanorods were obtained at a low temperature of 75°C, and the diameters increased with current density. Growth temperature seems to have a strong effect in generating well-defined hexagonal-shape nanorods with a smooth top edge surface. A film-like structure was observed for high current densities above -1.0 mA/cm^2^ and temperatures above 80°C due to the coalescence between the neighboring nanorods with large diameter. The nanorods grown at a temperature of 75°C with a low current density of -0.1 mA/cm^2^ exhibited the highest density of 1.45 × 10^9^ cm^-2^. X-ray diffraction measurements revealed that the grown ZnO crystallites were highly oriented along the *c*-axis. The intensity ratio of the ultraviolet (UV) region emission to the visible region emission, *I*_UV_/*I*_VIS_, showed a decrement with the current densities for all grown samples. The samples grown at the current density below -0.5 mA/cm^2^ showed high *I*_UV_/*I*_VIS_ values closer to or higher than 1.0, suggesting their fewer structural defects. For all the ZnO/graphene structures, the high transmittance up to 65% was obtained at the light wavelength of 550 nm. Structural and optical properties of the grown ZnO structures seem to be effectively controlled by the current density rather than the growth temperature. ZnO nanorod/graphene hybrid structure on glass is expected to be a promising structure for solar cell which is a conceivable candidate to address the global need for an inexpensive alternative energy source.

## Background

In recent years, the hybrid structures of zinc oxide (ZnO) nanostructures on graphene have attracted much attention because the nanostructures can offer additional functionalities to graphene for realizing advanced nanoscale applications [1,2]. This is due to the superior properties of nanostructures such as quantum confinement effects and high surface-to-volume ratio [3]. ZnO nanostructures on graphene on glass is expected to be a promising structure for solar cell which is a viable candidate to address the global need for an inexpensive alternative energy source [4]. Recently, ZnO has been shown to be able to enhance the power conversion efficiency of conjugated polymer-based solar cells [5,6]. Typically, the electrode of a solar cell is formed by the transparent conductive oxides such as fluorine-doped tin oxide (FTO) or indium tin oxide (ITO) deposited on glass [4,7]. However, FTO and ITO are expensive and non-flexible in contrast to graphene which is cheap and flexible. In ZnO/graphene/glass-based solar-cell-structure system, graphene is expected to act as an excellent conducting transparent electrode material [8,9] because of its extraordinary electrical, thermal, and mechanical properties including a carrier mobility exceeding 10^4^ cm^2^/Vs and a thermal conductivity of 10^3^ W/mK [10-13]. Interestingly, the direct growth of ZnO nanorods on graphene with high crystallinity and uniformity has also been reported in several literatures so far [1,2,14,15]. Therefore, the ZnO/graphene/glass system can be regarded as one of the most conceivable candidates for solar cell application.

The growth of ZnO nanostructures on graphene can be performed either by vapor-phase method [1,4,16] or by liquid-phase method [2,15,17,18]. In general, the former is likely to involve high temperature and is also considered as a high-cost method. Moreover, in many cases, the vapor-phase method requires oxygen gas. There is a possibility that graphene is oxidized or etched out during the growth of the ZnO nanostructures because the oxidation of graphene occurs at temperature as low as 450°C [19]. On the other hand, the liquid-phase method such as electrochemical deposition has advantages over the vapor-phase method because of its simplicity, low cost, and low process temperature [20]. In addition, this method gives good controllability of both growth rate and structure dimension. In this paper, we report the effects of current density and growth temperature on morphological, structural, and optical properties of ZnO structures grown on monolayer graphene/glass by electrochemical deposition without using any supporting reagent.

## Methods

**Figure 1:**
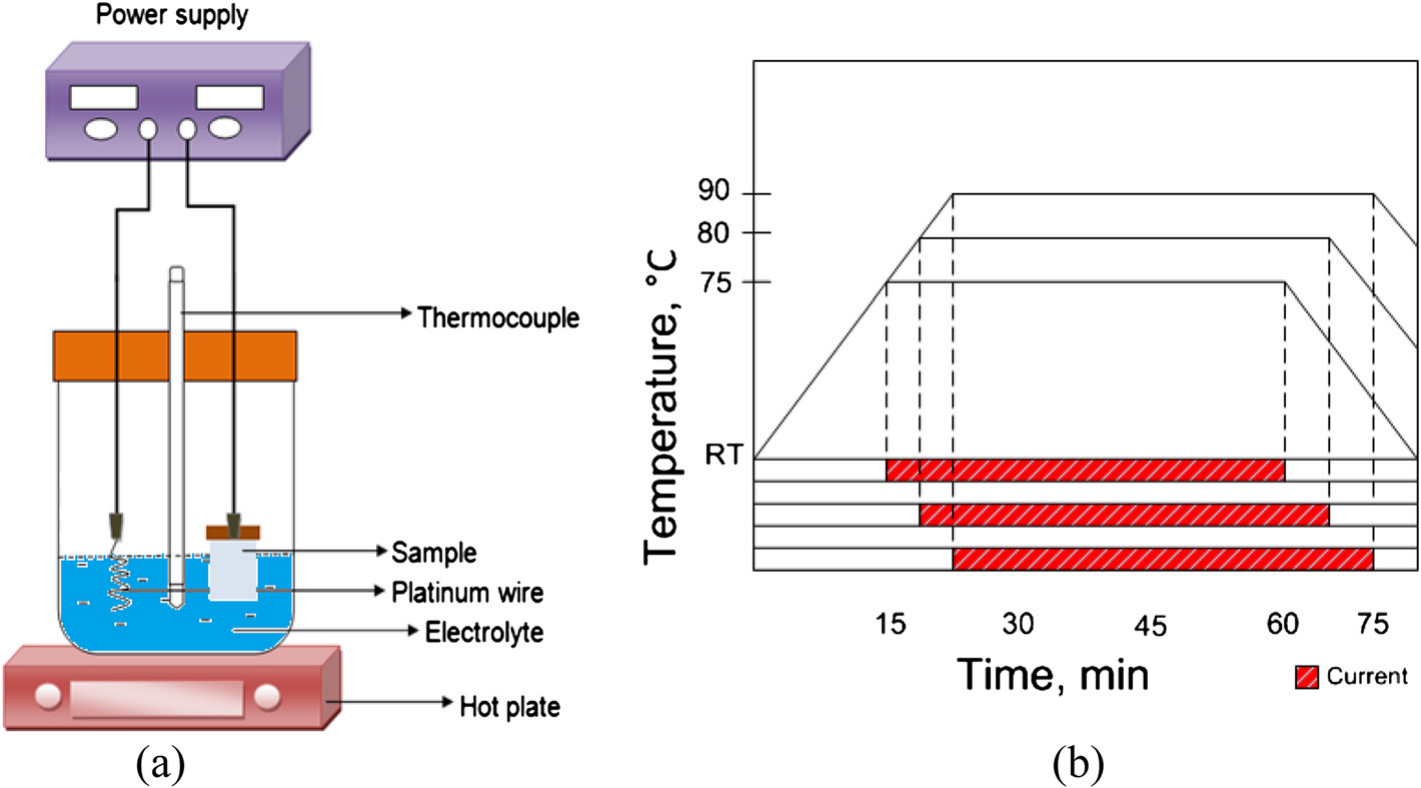
**Schematic diagram of the experimental setup and time chart. (a)** Schematic diagram of electrochemical deposition setup and **(b)** growth time chart.

A monolayer graphene on glass (Graphene Laboratories Inc., Calverton, NY, USA) was used as a substrate to grow ZnO nanostructures. The electrochemical deposition process was carried out using cathodic electrochemical deposition utilizing two electrodes of which a platinum (Pt) wire acts as an anode and a monolayer graphene as a cathode. The schematic diagram of the experimental setup is illustrated in Figure 1a. Zinc nitrate hexahydrate (Zn(NO_3_)_2_.6H_2_O) (Sigma-Aldrich (St. Louis, MO, USA), ≥99.0% purity) solution (10 mM) without any supporting agent was used as an electrolyte. The growth was done at current densities of -0.1, -0.5, -1.0, -2.0, and -3.0 mA/cm^2^ and temperatures of 75°C, 80°C, and 90°C for 45 min. The graphene/glass substrates were immersed into the electrolyte immediately after reaching the setting temperatures, i.e., 75°C, 80°C, and 90°C. The time chart of the growth is shown in Figure 1b. The grown structures were characterized using field-emission scanning electron microscopy (FESEM, Hitachi SU8030, Hitachi Ltd., Chiyoda-ku, Japan), energy dispersive X-ray (EDX) spectroscopy, X-ray diffraction (XRD, Rigaku RINT 2100, Rigaku, Shibuya-ku, Japan), photoluminescence (PL) spectroscopy (Horiba Jobin Yvon, Horiba Ltd., Tokyo, Japan), and UV–vis spectrometer (Cary 5000, Agilent Technologies, Inc., Santa Clara, CA, USA).

## Results and discussion

At first, it is worth to describe the chemical reactions that take place during the growth of ZnO structures on graphene [15]. As shown by Equation 1, Zn(NO_3_)_2_ decomposes to Zn ions (Zn^2+^) and nitrate ions (NO_3_ ^-^). Hydroxide ions (OH^-^) are produced during the reduction process of the water (H_2_O) with NO_3_ ^-^ ions (Equation 2). Then, OH^-^ reacts with Zn^2+^ to form the complex compound, Zn(OH)_2_ (Equation 3). Finally, the formed Zn(OH)_2_ continues to dihydrate into ZnO with the presence of heat. While at the anode, water is oxidized to produce hydrogen ions (H^+^).

Cathode:

(1) $$Zn{\left({\mathrm{NO}}_{3}\right)}_{2}\to \;{Zn}^{2+}{{+2\mathrm{NO}}_{3}}^{{}^{-}}$$

(2) $${{\mathrm{NO}}_{3}}^{{}^{-}}+\;H_{2}O+2e^{{}^{-}}\to \;{{\mathrm{NO}}_{2}}^{{}^{-}}+2{OH}^{{}^{-}}$$

(3) $${Zn}^{2+}+2{OH}^{{}^{-}}\to \;Zn{\left(OH\right)}_{2}$$

(4) $$Zn{\left(OH\right)}_{2}\to \;ZnO\;+\;H_{2}O$$

Anode:

(5) $$H_{2}O\;\to \;½\;O_{2}+2H^{+}+2e^{{}^{-}}$$

**Figure 2:**
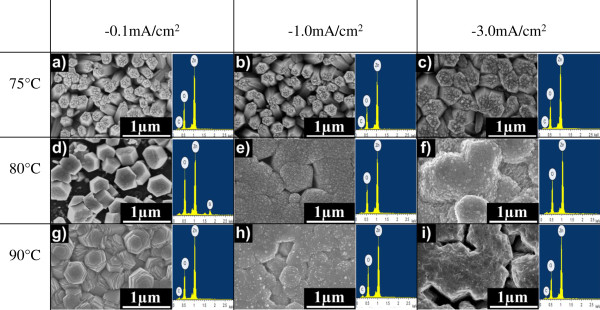
**FESEM images and EDX spectra of ZnO nanostructures.** Top view of FESEM images and EDX spectra of ZnO nanostructures grown with different current densities at temperatures of **(a-c)** 75°C, **(d-f)** 80°C, and **(g-i)** 90°C.

Figure 2 shows the top view FESEM images together with the corresponding EDX spectra of the grown ZnO structures on graphene at temperatures of 75°C, 80°C, and 90°C with current densities of -0.1, -1.0, and -3.0 mA/cm^2^. It can be seen that the morphologies of the structures seem to be strongly dependent on both applied current density and temperature. At the low temperature of 75°C, the growth of vertically aligned ZnO nanorods was observed as shown in Figure 2a,b,c. The diameters of the grown nanorods increase with the current densities. This is presumably due to the higher current density leading to a rapid formation of OH^-^ ions at the surface of graphene which then may result in much denser and larger seeds in the early stage of the growth process [14]. The grown nanorods show considerably good hexagonal-shape structure, but the top end surfaces of nanorods are likely to be rough. At the temperature of 80°C, it can be seen that well-defined hexagonal-shape nanorods with smooth top end surfaces were obtained for the current density of -0.1 mA/cm^2^. When the current density increases from -0.1 to -1.0 mA/cm^2^, the diameters of nanorods increase, resulting in the formation of film-like structures due to the coalescences between the neighboring nanorods with large diameter. Well-defined hexagonal-shape structures were also observed at temperature of 90°C for the low current density of -0.1 mA/cm^2^. These results suggest that a high growth temperature promotes the generation of the well-defined hexagonal shape of nanorods without using supporting reagents such as hexamethylenetetramine (HMTA) [2]. The coalescences between the neighboring nanorods observed on the samples grown at the current densities above -1.0 mA/cm^2^ are due to the increase in the diameters of nanorods. From the analysis of the EDX spectra, only zinc (Zn), oxygen (O) and carbon (C) elements were detected in all the grown samples and the total compositional atomic percentages of Zn and O were estimated to be above 95%. Table 1 summarizes the morphological structures, densities, and diameters of the grown nanostructures including comparison with other works which were also performed on graphene. In this study, the nanorods grown at the low temperature of 75°C with low current density of -0.1 mA/cm^2^ exhibited the highest nanorod density of 1.45 × 10^9^ cm^-2^. This value is one order higher than that of the work reported by Xu et al. [14]. Here, the nanorods were also grown using the similar technique, electrolyte, and current density but at a higher temperature of 90°C. Moreover, our highest nanorod density is two orders higher than that of nanorods grown in a mixture of zinc nitrate and HMTA by hydrothermal process as reported by Kim et al. [18]. Again, this density is in the same order with the nanorods grown in a mixture of zinc nitrate and HMTA at a bit higher current density of -0.5 mA/cm^2^ and a higher temperature of 80°C using an electrochemical deposition as reported by Aziz et al. [2]. Furthermore, our highest nanorod density was also found to be in the same order with nanorods grown at 800°C by thermal evaporation [1] and with nanoneedles grown at 400°C by metal-organic vapor-phase epitaxy (MOVPE) [16].

**Table 1:** Density and diameter of the grown ZnO nanorods

**Method**	**Temperature,°C**	**Current density, mA/cm**^**2**^	**Electrolyte/source**	**Density, cm**^**-2**^	**Diameter of nanorods, nm**	**Morphological structure**
Electrochemical deposition (this work)	75	-0.1	Zinc nitrate solution	1.45 × 10^9^	100 to 275	Nanorods
		-0.5^a^		8.09 × 10^8^	225 to 575	Nanorods
		-1.0		1.24 × 10^9^	100 to 325	Nanorods
		-2.0^a^		3.53 × 10^8^	200 to 825	Nanorods
		-3.0		7.47 × 10^8^	250 to 500	Nanorods
	80	-0.1		5.60 × 10^8^	243 to 500	Nanorods
		-1.0		-	-	Film-like structure
		-3.0		-	-	Film-like structure
	90	-0.1		6.22 × 10^8^	214 to 500	Nanorods
		-1.0		-	-	Film-like structure
		-3.0		-	-	Film-like structure
Electrochemical deposition [14]	90	-0.15	Zinc nitrate solution	5.83 × 10^8^	370 to 780	Nanorods
Electrochemical deposition [2]	80	-0.1	Zinc nitrate solution and HMTA	1.84 × 10^7^	190 to 450	Nanorods
		-0.5		1.37 × 10^9^	260 to 480	Nanorods
		-1.0		1.24 × 10^8^	660 to 1,000	Nanorods
		-1.5		3.42 × 10^7^	950 to 1,330	Nanocrystal
		-2.0		2.32 × 10^7^	570 to 2,030	Rods
Hydrothermal process [18]	60	-	Zinc nitrate solution and HMTA	3.10 × 10^7^	710	Nanorods
	80	-		3.00 × 10^7^	680	Nanorods
	95	-		4.20 × 10^7^	690	Nanorods
Thermal evaporation [1]	600	-	Zn powder and oxygen gas	-	-	Nanocluster
	800	-		6.86 × 10^9^	50 to 150	Nanorods
	1,000	-		-	-	Thin film
Metal-organic vapor-phase epitaxy (MOVPE) [16]	400	-	Diethylzinc (DEZn) and oxygen gas	4.00 × 10^9^	100 ± 10	Nanoneedles
	600	-		8.00 × 10^7^	90 ± 20	Nanoneedles
	750	-		5.00 × 10^7^	Not stated	Nanoneedles

^a^SEM data not shown.

**Figure 3:**
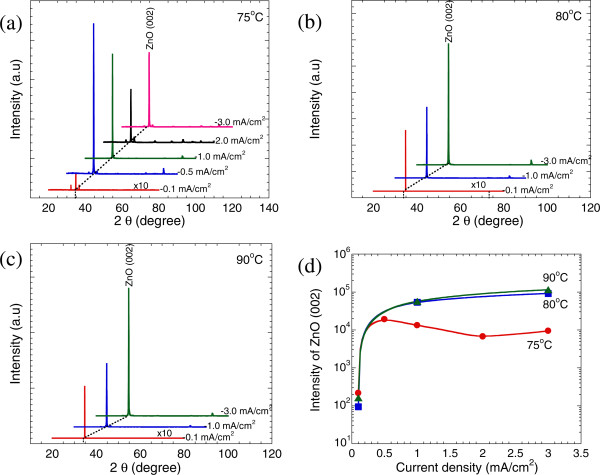
**XRD spectra of the grown ZnO structures with different applied current densities and temperatures.** XRD spectra of the grown ZnO structures with different applied current densities at temperatures of **(a)** 75°C, **(b)** 80°C, and **(c)** 90°C. **(d)** Current density vs. intensity of the ZnO (002) peak.

Figure 3a,b,c shows the XRD spectra of the nanorods grown at different current densities and temperatures. All observed reflection peaks can be indexed to the hexagonal wurtzite phase of ZnO (JCPDS card no. 36–1451). It can be clearly seen that the intensity of the (002) diffraction peak is much stronger than the other peaks for all samples indicating that these as-grown ZnO structures are highly oriented along the *c*-axis. Figure 3d shows the intensity of ZnO (002) as a function of current density for temperatures of 75°C, 80°C, and 90°C. It is well known that the intensity of the diffraction peak has a close connection with the thickness of the measured structure. Therefore, the reason why the intensities for the samples grown at 75°C are low and do not show significant changes for all current densities is probably their thin layer thicknesses or nanorod structures. For both temperatures of 80°C and 90°C, the intensity of the ZnO (002) peak increases drastically with the current density, suggesting that the thicknesses of grown structures are relatively thick and the thicknesses increase with the current density. However, the differences between the intensities of ZnO (002) for the sample grown at 80°C and those for the samples grown at 90°C are very small, suggesting the lesser effect of temperature in promoting the thicknesses of the grown structures.

**Figure 4:**
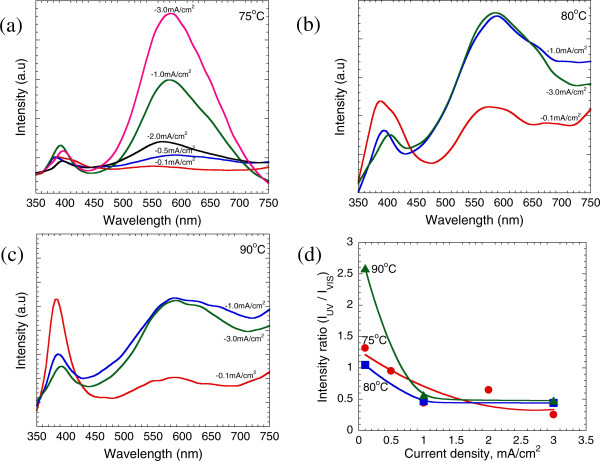
**PL spectra of the grown ZnO structures with different applied current densities and temperatures.** PL spectra of the grown ZnO structures with different applied current densities at temperatures of **(a)** 75°C, **(b)** 80°C, and **(c)** 90°C. **(d)** Current density vs. PL intensity ratio, *I*_UV_/*I*_VIS_.

The optical characteristics of ZnO were investigated using room temperature (RT) PL spectroscopy. Figure 4a,b,c shows the PL spectra of the ZnO nanostructures deposited on the graphene layers at temperatures of 75°C, 80°C, and 90°C with different applied current densities, respectively. Two distinct emission bands can be seen: one of which is located in the wavelength range of 382 to 402 nm of the ultraviolet (UV) region, and the other is located in the wavelength range of 565 to 589 nm of the visible region. This former is known as the near-band-edge (NBE) emission and could be referred to as an intrinsic property of the wurtzite crystal structure of ZnO and originated from the excitonic recombination [21]. On the other hand, the latter has been reported to be due to the radiative recombination of photon-generated holes with a single ionized charge of specific defects such as O vacancies or Zn interstitials [22]. Figure 4d summarizes the PL intensity ratio of the UV region emission to the visible region emission, denoted by *I*_UV_/*I*_VIS_, as a function of current density. Regardless of the difference in growth temperature, the *I*_UV_/*I*_VIS_ values decrease linearly with the increase in current density from -0.1 to -1.0 mA/cm^2^ for all temperatures, whereas those values are almost unchanged in the range from -1.0 to -3.0 mA/cm^2^. The *I*_UV_/*I*_VIS_ values for the samples grown at the current densities of -0.1 and -0.5 mA/cm^2^ are closer to or larger than 1.0. The highest ratio of 2.5 is obtained on the sample grown at the temperature of 90°C and current density of -0.1 mA/cm^2^. In general, the higher the *I*_UV_/*I*_VIS_ value, the fewer the structural defects in the grown structures [23,24].

**Figure 5:**
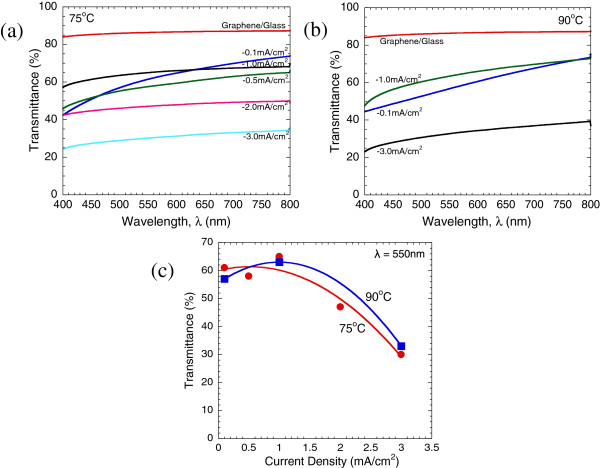
**Optical transmittance of the ZnO structures.** Optical transmittance of the ZnO structures grown at **(a)** 75°C and **(b)** 90°C with different applied current densities. **(c)** Current density vs. transmittance at wavelength of 550 nm.

Figure 5a,b shows the optical transmittance spectra of ZnO structures grown at 75°C and 90°C, respectively. Figure 5c shows the relation between the current density and the transmittance at the wavelength of 550 nm. The samples grown at low current densities (below -1.0 mA/cm^2^) exhibit high transmittance values up to 65% due to their thin structures, whereas those grown at high current densities show low transmittance values due to their thick structures. No significant difference between the samples grown at the low temperature of 75°C and those grown at the high temperature of 90°C seems likely to prove a lesser effect of temperature on the thickness. The highly transparent ZnO/graphene hybrid structure is expected to be applicable for the fabrication of solar cell device as well as for other kinds of transparent optoelectronic devices [13].

## Conclusions

The effects of current density and temperature on the morphological, structural, and optical properties of ZnO grown on graphene were studied. Vertically aligned nanorods were obtained at low growth temperatures. Growth temperature has a close connection with the appearance of the well-defined hexagonal-shape nanorods with smooth top end surface. A film-like structure caused by the coalescences between the neighboring nanorods with large diameters was observed on the samples grown at high current densities and high temperatures. The nanorods grown at a low temperature and low current density tended to exhibit a high density of nanorod. The XRD measurements revealed that the grown ZnO structures were highly oriented along the *c*-axis. The samples grown at low current densities seem to show fewer structural defects in the grown structures. The ZnO/graphene structures exhibited high transmittance values up to 65% at the light wavelength of 550 nm. ZnO nanorod/graphene hybrid structure on glass is expected to be a promising structure for solar cell which is a viable candidate to address the global need for an inexpensive alternative energy source.

## Competing interests

The authors declare that they have no competing interests.

## Authors’ contributions

NAH and HY designed and performed the experiments; participated in the characterization and data analysis of FESEM, XRD, PL and UV–vis; and prepared the manuscript. MRM participated in the PL and UV–vis characterization. TT participated in the XRD characterization and revision of the manuscript. AMH participated in the monitoring of the experimental work, data analysis, discussion, and revision of the manuscript. All authors read and approved the final manuscript.
